# TRPA1-dependent and -independent activation by commonly used preservatives

**DOI:** 10.3389/fphar.2023.1248558

**Published:** 2023-10-04

**Authors:** Maximilian L. Mager, Cosmin I. Ciotu, Markus Gold-Binder, Stefan Heber, Michael J. M. Fischer

**Affiliations:** Center for Physiology and Pharmacology, Medical University of Vienna, Vienna, Austria

**Keywords:** TRPA1, preservative, inflammation, sensitisation, pain, TRP channel, parenteral

## Abstract

**Background and purpose:** Addition of preservatives ensures microbial stability, especially in multidose containers of parenterally administered pharmaceuticals. These compounds can cause side effects, and particularly at the site of application, might elicit or facilitate pain. TRPA1 is a cation channel expressed in peripheral neurons which contributes to pain and inflammation and is sensitive to many irritants. The most commonly used preservatives, in particular with a focus on parenteral formulations, were investigated for their potential to activate TRPA1.

**Experimental approach:** Sixteen preservatives were screened for mediating calcium influx in human TRPA1-transfected HEK293t cells. Untransfected cells served as control, results were further validated in mouse sensory neurons. In addition, proinflammatory mediators serotonin, histamine and prostaglandin E2 were co-administered to probe a potential sensitisation of preservative-induced TRPA1 activation.

**Key results:** Butylparaben, propylparaben, ethylparaben, bronopol, methylparaben, phenylethyl alcohol and phenol induced a TRPA1-dependent calcium influx in transfected HEK293t cells at concentrations used for preservation. Other preservatives increased calcium within the used concentration ranges, but to a similar degree in untransfected controls. Serotonin, histamine, and prostaglandin enhanced TRPA1 activation of phenylethyl alcohol, bronopol, ethylparaben, propylparaben and butylparaben.

**Conclusion and implications:** Systematic screening of common preservatives applied for parenterally administered drugs resulted in identifying several preservatives with substantial TRPA1 channel activation. This activation was enhanced by the addition of proinflammatory meditators. This allows selecting a preservative without TRPA1 activation, particularly in case of pharmaceuticals that could act proinflammatory.

## 1 Introduction

The transient receptor potential ankyrin 1 (TRPA1) is a cation channel primarily expressed in sensory neurons (Bautista et al., 2013; Usoskin et al., 2015). TRPA1 is also found in other tissues, where it might well be of functional relevance. Therefore, TRPA1 is considered a potential target for the treatment of pain (Koivisto et al., 2018) but also for other diseases (Heber and Fischer, 2019). A principal function is to detect exogenous potentially damaging stimuli (Bandell et al., 2004; Bautista et al., 2006). TRPA1 has a high calcium and sodium conductance, elevating cytoplasmic calcium levels and generating action potentials in neurons (Jordt et al., 2004).

Propofol, for which intravenous injection will only generate limited extravasal levels, is immediately accompanied by pain in the majority of patients (Bakhtiari et al., 2021). The latter is caused by activation of TRPA1 (Fischer et al., 2010). Parenterally applied drugs can elicit pain or hyperalgesia. This includes local anaesthetics such as lidocaine, which also cause pain, at least partially involving TRPA1 (Leffler et al., 2008). In addition, for continuously applied prostacyclin mimetics, a slowly developing but frequently occurring and treatment-limiting hyperalgesia has been described (Picken et al., 2019).

The majority of TRPA1 activators are electrophiles, despite a variety of non-covalent agonists, including the chemically similar plant constituents menthol, thymol and carvacrol (Xu et al., 2006; Karashima et al., 2007; Lee et al., 2008), as well as nicotine (Talavera et al., 2009), clotrimazole (Meseguer et al., 2008), nifedipine (Fajardo et al., 2008) and nonsteroidal anti-inflammatory drugs (Hu et al., 2010). TRPA1 is characterised by its outstanding ability to detect reactive electrophile molecules, which covalently react with N-terminal cytoplasmic cysteine residues C621, C641, C665 and thus trigger calcium influx (Hinman et al., 2006; Bahia et al., 2016). This mediates external stimuli as the commonly used agonist allylisothiocyanate (Macpherson et al., 2007), but also endogenous stimuli such as unsaturated lipid mediators occurring in inflammation or methylglyoxal occurring in diabetes (Eberhardt et al., 2012; Ciardo and Ferrer-Montiel, 2017).

There are more than a hundred parenteral formulations on the market, which use multi-dose vials and therefore contain preservatives (Meyer et al., 2007). Among these, e.g., parabens, which are alkyl esters of p-hydroxybenzoate, cause specific activation of the TRPA1 channel in both heterologous expression systems and native sensory neurons (Fujita et al., 2007). The most commonly used parabens are methyl 4-hydroxybenzoate and propyl 4-hydroxybenzoate. These are used as preservatives not only in pharmaceuticals, but also in cosmetic products and foods due to their broad antibacterial properties, chemical stability and low toxicity (Aalto et al., 1953; Soni et al., 2002). Ortho-cresol, which is also widely used as a preservative, belongs to the class of substituted alkylphenols and activates TRPA1 (Lee et al., 2008). Preservatives allowed for parenteral formulations (Himoudy, 2016) have not been systematically investigated for TRPA1 activation, and in particular not with respect to the concentration range used for preservation (Otero-Losada, 1999; Boukarim et al., 2009; Kapupara et al., 2016; Lincho et al., 2021). TRPA1 activation is assumed to facilitate neurogenic inflammation and subsequently proinflammatory mediator release (Abdulkhaleq et al., 2018), which would further increase local sensitivity. Therefore, it was investigated whether prostaglandin E2, histamine and serotonin (5-HT) would enhance the sensitivity of TRPA1 to preservatives.

## 2 Materials and methods

### 2.1 Chemicals and solutions

Methylhydroxybenzoate, ethylhydroxybenzoate, m-cresol, propylhydroxybenzoate, butylhydroxybenzoate, sodium benzoate, chlorhexidine, benzoic acid, potassium sorbate, sorbic acid and cetylpyridinumchloride were obtained from Sigma-Aldrich (St. Louis, MI). Bronopol and phenylethyl alcohol were obtained from TCI Deutschland GmbH (Eschborn, Germany), benzalkonium chloride and benzethonium chloride from Fischer Scientific (Waltham, MA), chlorobutanol from MedChemExpress (NJ), benzyl alcohol from Biozol Diagnostica (Eching, Germany) and phenol from Boehringer Mannheim (Mannheim, Germany). Histamine was obtained from Fischer Scientific (Waltham, MA), Prostaglandin E2 from Adipogen (Füllinsdorf, Switzerland) and serotonin from Fluorochem (Hadfield, United Kingdom). Allyl isothiocyanate (AITC) was obtained from Fisher Scientific (Waltham, MA), capsaicin from Biotrend (Colonge, Germany). All substances were diluted in extracellular solution, composed of the following ingredients (in mM): 145 NaCl, 5 KCl, 10 glucose, 10 HEPES, 1.25 CaCl_2_, and 1 MgCl_2_ buffered to pH 7.4 with NaOH.

### 2.2 HEK293t cell culture, transfection and plate reader calcium assay

HEK293t cells were grown in Dulbecco’s Minimal Essential Medium (DMEM D5648, Sigma-Aldrich), supplemented with penicillin, streptomycin and L-glutamine (1% each, all from Lonza, Basel, Switzerland). HEK293t cells were transfected with human TRPA1 (hTRPA1) using jetPEI transfection reagent (Polyplus, Illkirch, France). The plasmid was obtained from Paul Heppenstall and cloned into a pcDNA3.1 vector (Heber et al., 2019; Meents et al., 2019). The cells were then spread on poly-D-lysine coated black 96 well plates (∼30.000 cells/well) and incubated overnight at 37°C and 5% CO_2_.

The microfluorimetry of cytosolic calcium levels was performed as described before (Luo et al., 2011, p. 3). Briefly, cells were loaded with calcium 6 (Calcium 6 kit, Molecular Devices, San Jose, CA) in extracellular solution for 2 h according to the manufacturer’s protocol. Cells were not washed as fluorescence of the extracellular dye is absorbed by a dark dye in the kit. A pipetting fluorescence plate reader (FlexStation 3, Molecular Devices, San Jose, CA) was used to measure the change in intracellular Ca^2+^ concentration in hTRPA1 transfected and non-transfected HEK293t cells. Calcium 6 fluorescence, which was excited every 2 s at 485 nm, served as an index of intracellular calcium levels. Using a pre-set protocol, 50 µL of the assay solution was automatically pipetted onto the cells in 100 µL of medium. Fluorescence change is reported normalised to baseline fluorescence (dF/F0). Assays were performed at 37 °C (Heber et al., 2019). For experiments with histamine, PGE_2_ and 5-HT, these mediators were mixed with the preservatives and co-applied onto the cells.

### 2.3 Sensory neuron culture and single cell calcium assay

Breeding, euthanasia and all procedures of animal handling were performed according to regulations of animal care and welfare. Experiments were carried out in accordance with the European Communities Council Directive of 24 November 1986 (86/609/EEC). Wild type C57BL/6 or TRPA1^−/−^ mice were anaesthetised by an exposure to isoflurane or rising CO_2_ levels and euthanized by cervical dislocation. DRGs from all spinal levels were excised and transferred to DMEM containing streptomycin, penicillin and L-glutamine, treated with 1 mg·mL^−1^ collagenase (Sigma-Aldrich) and 3 mg·mL^−1^ Dispase II (Roche) for 55 min at 37 °C. DRGs were then mechanically dissociated with a Pasteur pipette, centrifuged at 1,200 rpm for 5 min and plated onto 12 mm glass coverslips previously coated with poly-D-lysine (100 µg·mL^−1^, Sigma-Aldrich). Dorsal root ganglia neurons were cultured in DMEM supplemented with streptomycin, penicillin and L-glutamine and mouse nerve growth factor 100 ng·mL^−1^ (Alomone Labs, Tel Aviv, Israel) at 37 °C and 5% CO_2_ for 15–30 h.

The coverslips were incubated with 3 µM Fura-2 AM (Biotium, CA, United States) for 30 min at 37°C and 5% CO_2_, before placement in 35 mm glass-bottom dishes in extracellular solution. After 10 min, allowing removal or cleaving the ester of the dye, dishes were mounted onto an inverted microscope (IX73, Olympus, Tokyo, Japan) and imaged using a ×10 objective. Cells were continously superfused with extracellular solution using a software-controlled 8-channel, gravity-driven, common-outlet system (ALA Scientific Instruments Inc., NY, United States). This superfusion was switched to different solutions after baseline recording. After exposure to the preservative, TRPA1 agonist AITC and TRPV1 agonist capsaicin were applied. This allowed to calculate for single neurons a correlation of the response to the preservative and a subsequent stimulus, using a product-momentum correlation. Finally, a positive control detecting viable cells was added at the end of each recording, using depolarization by an extracellular solution with 60 mM KCl (isotonic replacement of NaCl).

Fura-2 was alternatingly excited for 30 ms by a 340-nm LED (50 mW, used at 100%) and by a 385-nm LED (1,435 mW, used at 5%) by an Omicron LEDHub (Laserage-Laserprodukte GmbH, Rodgau-Dudenhofen, Germany). Fluorescence emission was long-pass filtered at 495 nm, and pairs of images were acquired at a rate of 1 Hz with a 5.76-megapixel 16-bit CCD camera (6.5-μm pixel edge length, 22-mm sensor diagonal, Kinetix 22; Teledyne Photometrics AZ, United States). The hardware was controlled by the μManager 1.4 plugin in ImageJ (Schneider et al., 2012). The background intensity was subtracted before calculating the ratio between the fluorescence emitted when the dye was excited at 340 nm and at 385 nm (F340/F385 nm). The time course of this ratio was analysed for regions of interest adapted to individual cells.

### 2.4 Statistical analysis

**FIGURE 1:**
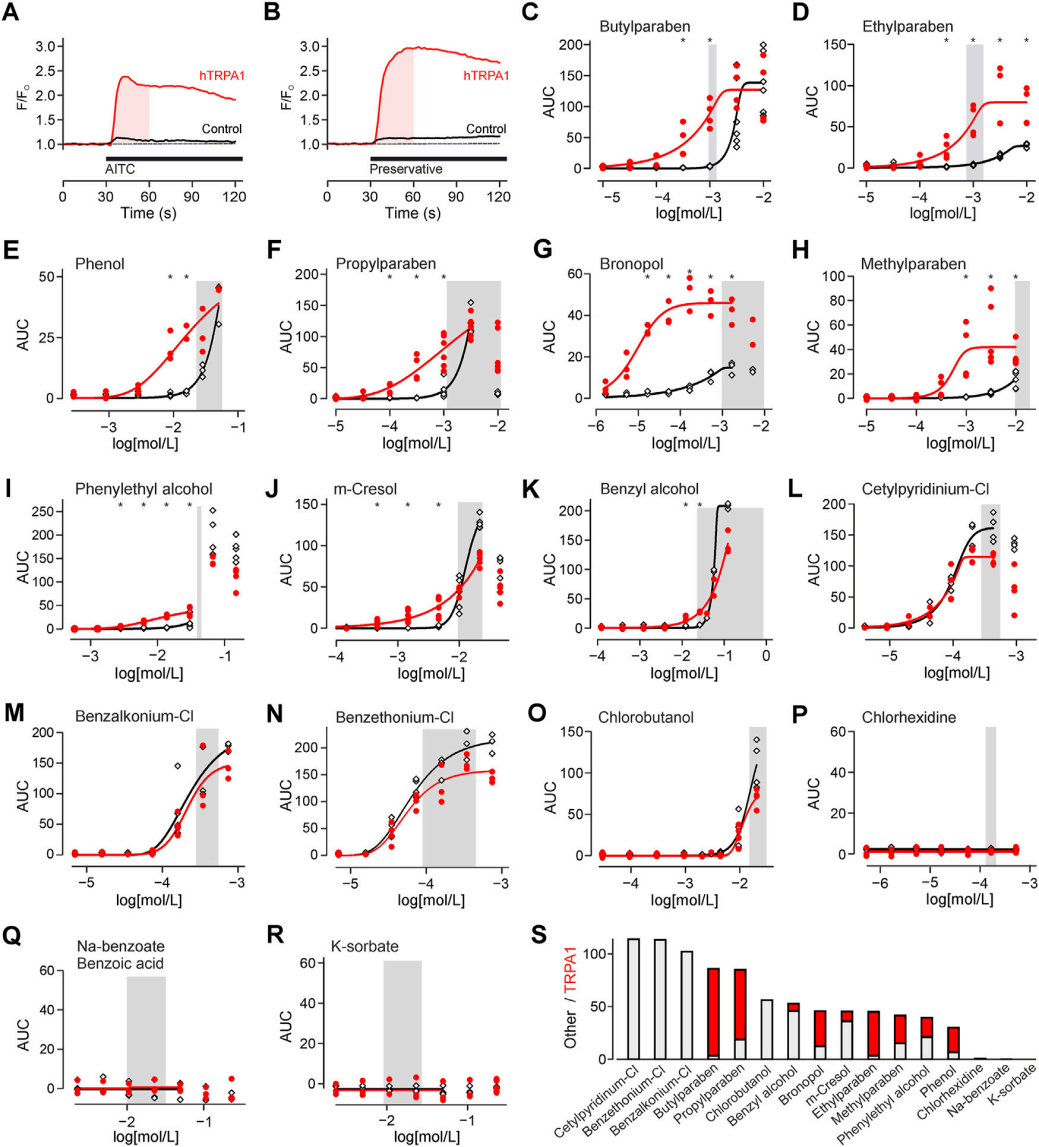
Preservatives for parenteral formulation activate TRPA1. **(A)** Experimental protocol on the fluorescence plate reader. Substances were automatically added after 30 s. The shaded area indicates the area under the curve (AUC) analysed in all further experiments. In this experiment TRPA1 agonist allylisothiocyanate 25 µM (AITC) is used as positive control. This causes a response in hTRPA1 transfected (red trace), but not in untransfected HEK293t cells (black trace). Traces are the mean of five repetitions, normalized to the 30 s before application. **(B)** Identical experiment with application of a preservative, in this panel bronopol 50 µM. Traces are the mean of five repetitions. **(C-R)** Concentration-response of the indicated preservative in hTRPA1-transfected and untransfected HEK293t cells. Shaded area reflects the typical range in clinical use. The panels are sorted from high to no TRPA1 activation at the lower end of the commonly used preservatives concentration. *indicates a significantly larger response in hTRPA1-transfected compared to untransfected cells. **(S)** Activation by the preservatives at the left border of the shaded concentration range. In contrast to the focus on TRPA1 in the other panels, the bar chart was sorted by total activation. The stacked histogram visualizes the TRPA1-independent (grey) activation, and the additional TRPA1-dependent (red) activation on top.

An area under the curve (AUC) was calculated for 30 s after application in HEK293t cells, as these respond rapidly and show a high level of TRPA1 expression. In contrast, to detect also slower but steady rises in calcium, a longer area under the curve of 60 s was used of for sensory neurons. As baseline for AUC calculations, the arithmetic mean of the 30 s before application were used. Whether preservatives activate TRPA1 was assessed using dose-response curves with the concentration of a preservative as predictor and the AUC value described above as the dependent variable. For each preservative, a 3-parameter dose response curve with the parameters bottom, EC_50_ and top was fitted. In case residuals were not approximately normally distributed, or fit parameters were unstable, a 4-parameter dose response curve containing the Hill slope as a fourth parameter was fitted. In case this also did not result in adequately distributed residuals or unstable parameters, a 5-parameter dose response curve was fitted, which includes an asymmetry factor as a fifth parameter. When the most adequate type of dose response curve was not apparent, the best model was chosen based on Akaike’s information criterion. Whether responses to preservatives in TRPA1-transfected were above naïve cells was tested by unpaired student’s t-tests. For aggregation of results in Figure 1S, the response at the lowest commonly used preservative concentration was taken from the experiment, or interpolated from the curve fit.

Sensitization of TRPA1 by inflammatory mediators was assessed using a mixed linear model. The preservative concentration and the inflammatory mediator were used as a factor. A random factor accounted for the dependency of values derived from the same 96 well plate. To stabilise the residual distribution, data were transformed prior to the analysis using log [x - minimum(x) + 1]. No adjustment for multiplicity was performed. *p*-values ≤0.05 were considered statistically significant. Curve fittings and graphs were generated by GraphPad 9, the mixed linear model was estimated by IBM SPSS statistics 29.

## 3 Results

TRPA1 has a high calcium ion conductance, therefore activation was monitored by calcium imaging. For TRPA1 agonist AITC, activation was probed by comparing hTRPA1-transfected to untransfected HEK293t cells (Figure 1A). With the same protocol, TRPA1 activation was probed for preservatives, in commonly used concentrations (Supplementary Table S1; Figure 1B). Four types of responsiveness to preservatives were observed. First, there were preservatives which caused only a TRPA1-dependent response, but none in control cells (Figures 1C, D). Second, there were preservatives with a TRPA1-dependent but also a smaller TRPA1-independent response (Figures 1E–I). Third, there were preservatives, which elevated cytosolic calcium in the relevant concentration range, but primarily in a TRPA1-independent manner (Figures 1J–O). Finally, there were preservatives which did not elevate intracellular calcium in the relevant concentration range (Figures 1P–R). To allow choosing preservatives by activation these were also sorted by total activation, cumulating the TRPA1-dependent and independent component (Figure 1S).

**FIGURE 2:**
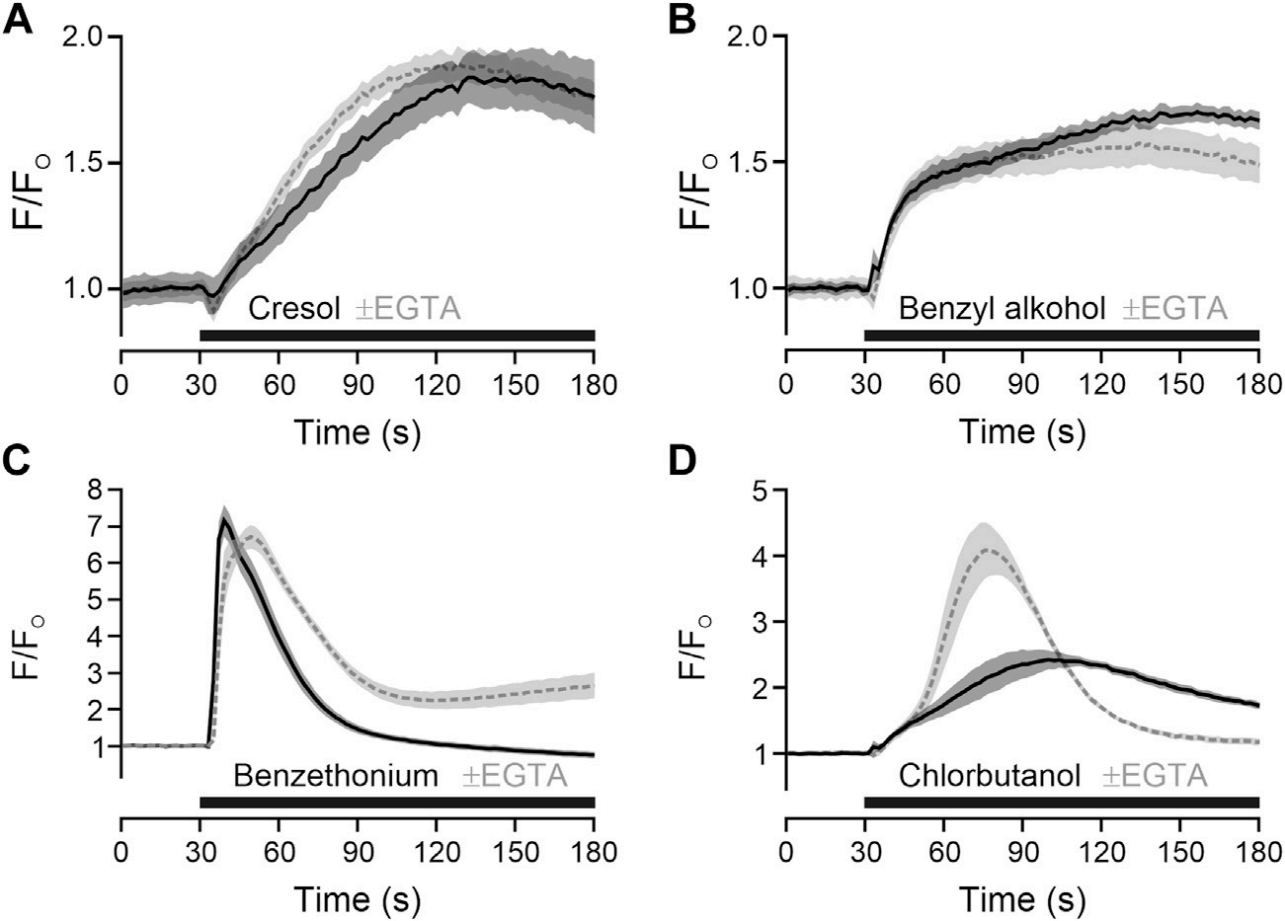
Preservatives with TRPA1-independent action release intracellular calcium. **(A)** m-Cresol 9.52 mM was applied (black bar) in the in presence of extracellular calcium (solid black line) or in absence of extracellular calcium and presence of EGTA 6.66 mM (dashed grey line). **(B)** Benzyl alcohol 48.1 mM, **(C)** benzethonium 0.09 mM and **(D)** chlorobutanol 15.5 mM were tested with the same approach. Traces represent the mean, shaded areas the standard error of the mean.

To add mechanistical insight, the source of calcium for TRPA1-independent activation was explored. For m-cresol and benzyl alcohol, responses in presence of extracellular calcium and responses in absence of extracellular calcium and presence of EGTA were largely similar, indicating a release from intracellular stores (Figures 2A, B). For benzethonium and chlorobutanol there were differences in the time courses (Figures 2C, D), nevertheless indicating that a large fraction of calcium came from intracellular stores.

**FIGURE 3:**
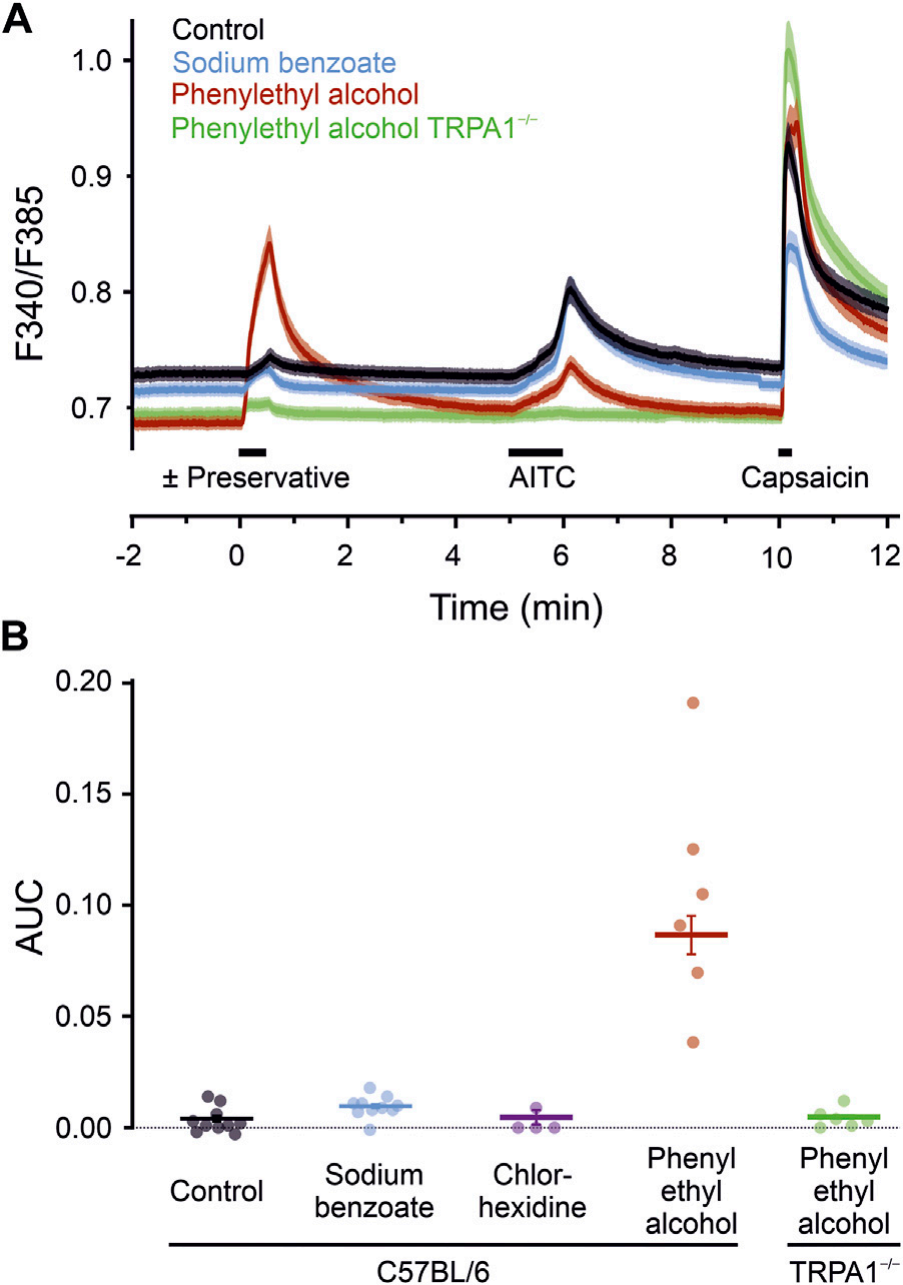
Preservatives for parenteral formulation activate sensory neurons. **(A)** Phenylethyl alcohol 30 mM induced a substantial increase in intracellular calcium (red trace, *n* = 954), compared with controls (black trace, *n* = 1,190), or sodium benzoate 10 mM (blue trace, *n* = 942). Sensory neurons of TRPA1^−/−^ mice (green trace, *n* = 773) showed a complete reduction in phenylethyl alcohol induced activation. Black bars indicate substance application. After a preservative, TRPA1 agonist AITC 100 µM and capsaicin 1 µM was applied. Traces represent the mean, shaded areas of the same colour the standard error of the mean. **(B)** Area under the curve calculated over a 60 s interval starting with the preservative application. Dots represent the means of the dishes. Horizontal bar and variance indicates mean ± SEM (395–1701 cells), tested on independent experimental days. Chlorhexidine 300 µM served as an additional preservative without neuronal activation. In contrast, calcium-elevation by phenylethyl alcohol is fully TRPA1-dependent, as it is absent in sensory neurons lacking the ion channel.

To test whether the results from the expression system translate to mammalian neuronal cells, cultured mouse DRG neurons were exposed to different preservatives. The calcium responses upon application of the test compounds tested were compared to the control condition (one-way ANOVA, F = 94, *p* < 0.001). In neurons from C57BL/6 wild type mice, sodium benzoate 10 mM and chlorhexidine 300 µM did not elicit responses (Figure 3). Phenylethyl alcohol 30 mM also elicited a substantial calcium response (*p* < 0.001 vs. control). These experiments allow sequential application and to correlate the response of a single neuron to further agonists. Responses to phenylethyl alcohol were positively correlated with those subsequently elicited in the same neurons by AITC (Pearson correlation coefficient *r* = 0.41, *n* = 954, *p* < 0.001, and *r* = 0.35, *p* < 0.001 for the 322 cells which responded to either phenylethyl alcohol or AITC). Phenylethyl alcohol might have caused some cross-desensitisation of AITC responsiveness, underestimating the correlation. The acute effects of phenylethyl alcohol observed in wildtype neurons were absent in neurons from TRPA1^−/−^ mice (*p* < 0.001 vs. wildtype, n.s. vs. control), indicating complete TRPA1-dependence.

**FIGURE 4:**
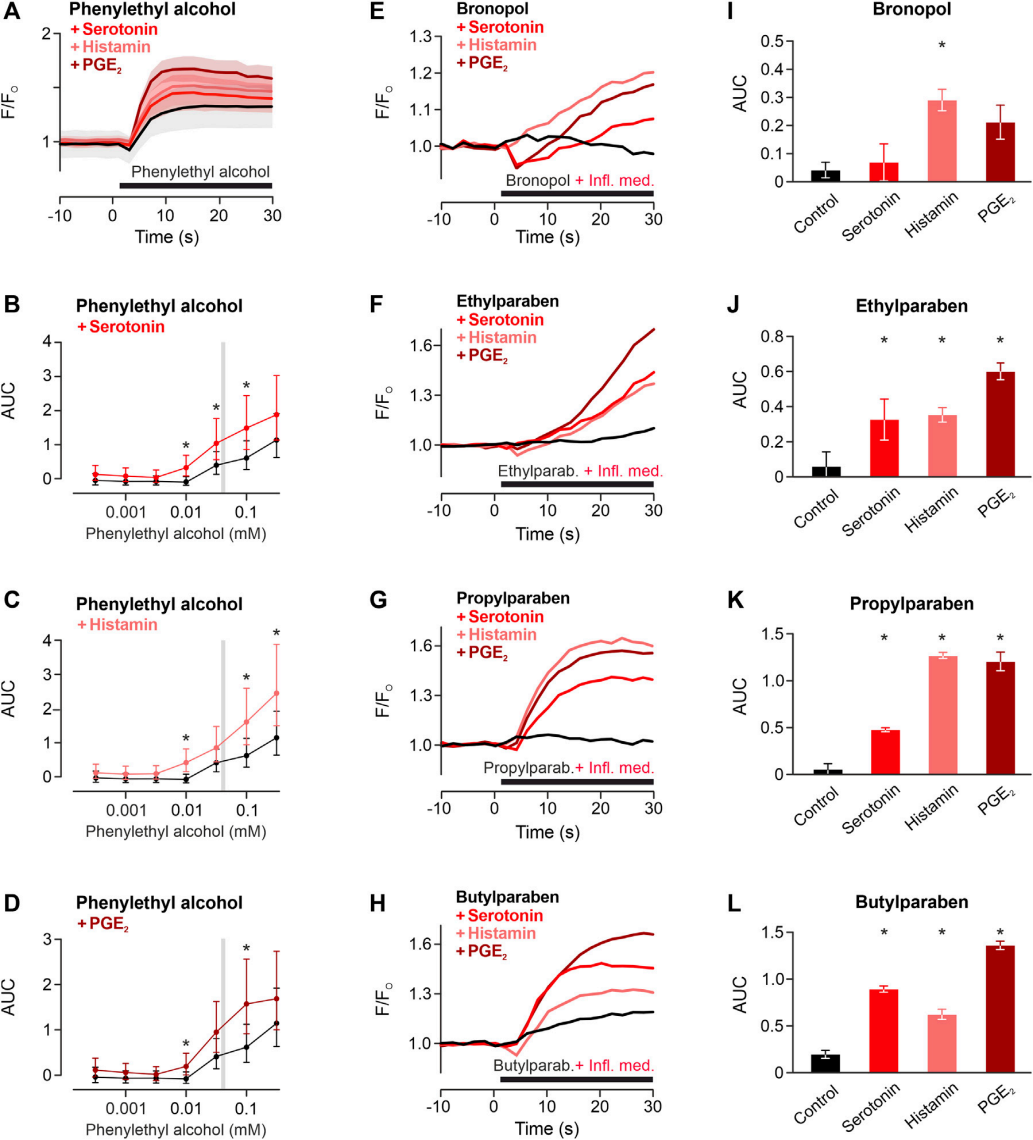
Inflammatory mediators sensitise TRPA1 activation of phenylethyl alcohol. **(A)** TRPA1-activating phenylethyl alcohol 30 mM applied alone (black) or co-applied with histamine 1 μM, serotonin 1 µM or prostaglandin E2 1 µM (PGE_2_). Traces are calcium-dependent fluorescence, normalised to the last 10 s before application, data are mean ± standard error of the mean. **(B–D)** Areas under the curve for phenylethyl alcohol around the typical range were higher in the presence of inflammatory mediators compared to control. Data are geometric least squares means with 95% confidence intervals estimated from a linear model. * indicates *p* < 0.05. Typical concentrations of four further preservatives for subcutaneous formulation tested for sensitisation of the TRPA1 response by inflammatory mediators (Infl. med.). **(E)** Bronopol 1.8 µM, **(F)** ethylparaben 100 μM, **(G)** propylparaben 100 μM, **(H)** butylparaben 100 µM were tested alone or coapplied with inflammatory mediators. Each trace represents the mean of three experiments, normalised to the last 10 s before application. **(I–L)** Area under the curve for experiments in panels **(E–H)**. Area under the curves of the 30 s application period. Data are mean with standard error of the mean. * indicates *p* < 0.05 vs. control.

Local inflammation generates inflammatory mediators. To investigate whether this might facilitate the TRPA1-dependent activation, the TRPA1-activating phenylethyl alcohol was applied to cells preincubated with the proinflammatory mediators histamine, serotonin and prostaglandin E2. In the TRPA1-dependent concentration range of phenylethyl alcohol, inflammatory mediators increased the TRPA1-dependent response (Preservative * inflammatory mediator *p* < 0.001, Figures 4A–D). For four further preservatives, one typical preservative concentration was applied with or without inflammatory mediators to hTRPA1-expressing HEK293t. Similar to phenylethyl alcohol, also bronopol, ethylparaben, propylparaben and butylparaben were sensitised by histamine, serotonin and prostaglandin E2 (One-way ANOVAs, F = 5.5, *p* = 0.024, F = 8.0, *p* = 0.0087, F = 89, *p* < 0.0001, F = 120, *p* < 0.0001 respectively, Figures 4E–L).

## 4 Discussion

A systematic investigation of sixteen preservatives demonstrated that several of these activate solely or predominantly through TRPA1 at a typically used concentration. Proinflammatory mediators histamine, serotonin, and prostaglandin E2 sensitised the preservative-induced activation of TRPA1.

Parenterally applied drugs for repeated withdrawal from multidose vials usually contain preservatives to prolong their usability. There are many aspects in choosing a preservative (Meyer et al., 2007). Further guidance was provided in reviews by David Elder and Patrick Crowley in American Pharmaceutical Review (2017). This includes consideration of biocidal activity, including pH-dependence and differential effects on bacteria, yeasts and mould, solubility, adsorption, stability, compatibility with the drug (e.g., interaction with large peptides as monoclonal antibodies) and side effects as mutagenicity and irritancy.

Given relevant species differences, first detected for menthol (Xiao et al., 2008), the present study investigated the human TRPA1 channel. Effects of preservatives on TRPA1 can be divided into three groups: no activation, activation above the target concentration, and unavoidable activation. The latter might still be minimised by using the lowest preserving concentration. For some of the approved preservatives, this confirms results of prior studies.

### 4.1 Preservatives causing TRPA1-dependent activation

TRPA1 activation has already been demonstrated for the widely used parabens such as methylparaben, ethylparaben, propylparaben and butylparaben as well as for o-cresol (Fujita et al., 2007; Startek et al., 2021). This includes evidence for pain-related behaviour elicited in mice by methylparaben (Fujita et al., 2007). In the present study, activation of human TRPA1-transfected HEK293t by methylparaben was not far from the 4.4 mM reported before, and the minor difference might be well explained by non-identical experimental settings (Fujita et al., 2007).

Phenol and alkylated phenols are non-electrophilic TRPA1 agonists (Startek et al., 2021), their antimicrobial mechanism was suggested to rely on enzyme inhibition (Denver et al., 2011). Phenol has not been reported to be painful, although it is used at high concentrations for neurolysis in cancer patients (Koyyalagunta et al., 2016). An additional local anaesthetic effect was hypothesized to explain the lack of pain upon phenol injection (Zamponi and French, 1993). Bronopol and phenylethyl alcohol have not been previously investigated for human TRPA1 activation. Intraperitoneal bronopol injection was described to cause an acute ‘transitory aggressive behaviour in rats’, which was described as ‘painful state’ (Nieminen and Möttönen, 1975). Importantly, even the lowest concentration used for preservation caused TRPA1 activation. For bronopol, a reaction with bacterial thiols and disulfide formation has been described as a mechanism of antimicrobial action (Shepherd et al., 1988). This fits well with the covalent action on N-terminal TRPA1 cysteines. In contrast, for phenylethyl alcohol bacterial membrane alterations have been reported as a mechanism of action (Corre et al., 1990). However, an irritant action of phenylethyl alcohol 6 g/L added to eye drops has been described in a blinded experiment in six subjects (Boer, 1981). Meta-cresol is a known irritant, with a TRPA1-dependent activation at low concentrations, at preserving concentrations there is also a TRPA1-independent component. Different formulations of growth hormone showed that pain perception was more for 0.25% m-Cresol compared to 0.9%–1.5% benzyl alcohol (Kappelgaard et al., 2004).

### 4.2 Preservatives causing TRPA1-independent activation

Benzyl alcohol, cetylpyridinium chloride, benzalkonium chloride and benzethonium chloride caused TRPA1-independent calcium influx at target concentrations. Chlorobutanol induced calcium influx at the highest tested concentration, however, maximum solubility in extracellular solution was below the preserving range. The cellular mechanism of the TRPA1-independent calcium influx in HEK293t cells is unknown. However, extracellular calcium was not required to observe the calcium elevation caused by TRPA1-independent preservatives. This indicates a ubiquitous intracellular calcium source, like the endoplasmic reticulum or mitochondria.

More pain was reported after shoulder arthroscopy by patients with benzyl alcohol in the saline solution compared to only saline, indicating the choice of preservative matters (Storey et al., 2014). Paravasation of intravenous drugs is underdocumented, and although cytotoxic, proinflammatory and irritant reactions are known for many pharmaceuticals, a potential contribution of preservatives to that is unknown (Le and Patel, 2014). Benzalkonium instillation into the bladder causes pain (Xu et al., 2011). No literature suggesting nociceptor activation, pain-related behaviour in animals or reports of pain was found for cetylpyridinium, benzethonium and chlorobutanol.

### 4.3 Preservatives for avoiding cellular activation

Importantly, there are substances available which do not activate at the required concentration for preservation. Potassium sorbate, sodium benzoate, and chlorhexidine elicited no calcium influx in untransfected HEK293t cells at any of the test concentrations.

A database search for these three substances did not yield literature suggesting nociceptor activation or induction or facilitation of pain-related behaviour in animals. Therefore, these substances are favourable for avoiding cell activation.

### 4.4 Inflammatory sensitisation

Inflammatory conditions alter neuronal sensitivity, and this includes TRPA1 expression and responsiveness (Bautista et al., 2013). Parenteral drugs are rarely deliberately applied to inflamed tissues, but, e.g., for application of a proinflammatory drug, the induced inflammation and the preservative might cause an additive or supra-additive effect. In inflammation, several proinflammatory mediators are upregulated (Choi and Di Nardo, 2018). This has led to the common uses of histamine, serotonin prostaglandin E2 and bradykinin as an ‘inflammatory soup’ to mimic the biological changes in inflamed tissue. In the *in vitro* model, bradykinin was omitted, as it elevated intracellular calcium in HEK293t, as previously demonstrated (Kramarenko et al., 2009). Therefore, sensitisation cannot be easily quantified or might be obfuscated by cross-desensitisation. HEK293t cells express the respective receptors for proinflammatory mediators (Atwood et al., 2011). Histamine, serotonin or prostaglandin E2 alone did not cause a calcium influx, but sensitised the preservative-induced TRPA1 activation. For phenylethyl alcohol 10 μM, a supra-additive response was observed, as neither the preservative nor the inflammatory mediators alone cause a response. Sensitisation by inflammatory mediators was confirmed for bronopol, ethylparaben, propylparaben, and butylparaben.

### 4.5 Limitations

The chosen substances reflect the currently used preservatives. Allowed substances may differ based on regulation of countries or regions and are subject to change. Further, some substances have been used in parenteral formulations but are currently only found in ophthalmic, nasal or otic formulations, or in creams, which applies, e.g., to phenylethyl alcohol, ethylparaben or butylparaben. Other substances, e.g., organomercury compounds like thiomersal have not been investigated, as their use in new parenteral formulations is rather limited. The presented experiments were mostly done on HEK293t cells and partially confirmed in sensory neurons; other cell types might have different sensitivity. Further, validation *in vivo* seems a necessary next step to judge the clinical relevance of the presented results.

### 4.6 Conclusion

Several preservatives commonly used in therapeutics activate TRPA1. This is particularly important in acute inflammation, where sensitization may add to hyperalgesia. The findings can serve as an orientation for the selection of preservatives to reduce TRPA1-dependent and independent activation.

## Data Availability

The raw data supporting the conclusion of this article will be made available by the authors, without undue reservation.
