# Supplementary material for: TRPA1-dependent and -independent activation by commonly used preservatives
Source: Front Pharmacol. 2023 Oct 4;14:1248558. doi: 10.3389/fphar.2023.1248558 (PMC10582264; doi:10.3389/fphar.2023.1248558)
Supplement: Supplementary file 1 [file Table1.DOCX]

Supplementary Material

TRPA1 activation by commonly used preservatives

Maximilian L. Mager, Cosmin I. Ciotu, Markus Gold-Binder, Stefan Heber, Michael J.M. Fischer*

*** Correspondence:** [michael.jm.fischer@meduniwien.ac.at](mailto:michael.jm.fischer@meduniwien.ac.at)

| Preservative | Concentration (mM) | Source |
| --- | --- | --- |
| Butylparaben | 0.95 - 1.3 | Lincho et al., 2 |
| Propylparaben | 1.2 - 11.8 | Lincho et al., 1 |
| Ethylparaben | 0.75 - 1.5 | 4 |
| Bronopol | 0.96 - 9.6 | Kapupara et al. |
| Phenylethyl alcohol | 42 | 2 |
| Methylparaben | 9.6 - 19 | Boukarim et al., Lincho et al., 1 |
| Phenol | 23 - 57 | 1 |
| m-Cresol | 9.5 - 23.8 | 1 |
| Benzalkonium (chloride) | 0.28 - 0.55 | 2 |
| Benzethonium (chloride) | 0.09 - 0.45 | 1 |
| Benzyl alcohol | 48 - 962 | 1, 2 |
| Chlorobutanol | 15 - 31 | 1 |
| Cetylpyridinium (chloride) | 0.28 - 0.55 | 1 |
| (potassium) sorbate | 9.1 - 18 | Boukarim et al., 1 |
| Benzoic acid / benzoate | 10 - 31 | Boukarim et al., 1 |
| Chlorhexidine | 0.13 - 0.21 | 1 |

**Supplementary Table 1.** **Concentration range for preservatives.** Inflammatory mediators sensitise TRPA1 activation by common preservatives. The International Council for Harmonisation of Technical Requirements for Pharmaceuticals for Human Use (ICH) recommends that hold time studies simulate the use in practice and provide ‘a 30-day in-use period is normally considered acceptable’ (Q1F Stability Guideline). The minimum effective amount of preservative should be used and evidence for effectiveness at the end of the intended usage period has to be provided separately for every drug formulation. In line with this approach, the regulatory authorities do not provide a range for preservative content. Therefore, the respective ranges are recommendations, which are based on general experiments and on available drugs. The last column provides the respective sources. Substances are sorted as in Figure 1, according to their induced cytosolic calcium responses. 1 https://compoundingtoday.com/Preservative/ 2 https://www.lfatabletpresses.com/articles/preservatives-concentration-dosage-pharmaceutical-liquid-preparation, 3 https://pharmacentral.com/learning-hub/ingredient-spotlight/benzalkonium-chloride/ 4 Based on relative antimicrobial action compared to other parabens
